# Multi‐omic profiling of squamous cell lung cancer identifies metabolites and related genes associated with squamous cell carcinoma

**DOI:** 10.1002/1878-0261.70121

**Published:** 2025-09-03

**Authors:** Johan Staaf, Daniel Ehinger, Hans Brunnström, Mats Jönsson, Frida Rosengren, Marija Kotevska, Anna Karlsson, Mattias Aine, Christian Frezza, Maria Planck, Elsa Arbajian

**Affiliations:** ^1^ Division of Oncology, Department of Clinical Sciences Lund Lund University Sweden; ^2^ Division of Translational Cancer Research, Department of Laboratory Medicine Lund University Sweden; ^3^ Department of Genetics, Pathology, and Molecular Diagnostics Skåne University Hospital Lund Sweden; ^4^ Division of Pathology, Department of Clinical Sciences Lund Lund University Sweden; ^5^ Department of Respiratory Medicine and Allergology Skåne University Hospital Lund Sweden; ^6^ Faculty of Medicine and University Hospital Cologne, Institute for Metabolomics in Ageing, Cluster of Excellence Cellular Stress Responses in Aging‐Associated Diseases (CECAD) University of Cologne Germany; ^7^ Faculty of Mathematics and Natural Sciences, Institute of Genetics, Cluster of Excellence Cellular Stress Responses in Aging‐Associated Diseases (CECAD) University of Cologne Germany

**Keywords:** creatine, lung cancer, metabolomics, SLC6A8, squamous cell lung carcinoma

## Abstract

Squamous cell lung carcinoma (SqCC) is the second most common histological subtype of lung cancer. Besides tumor‐initiating and promoting DNA, RNA, and epigenetic alterations, aberrant cell metabolism is a hallmark of carcinogenesis. This study aimed to identify SqCC‐specific key regulators that could eventually be used as new anticancer targets. Transcriptional and metabolomic data were gathered for a cohort of resected lung cancers. SqCC‐specific differentially expressed genes were integrated with metabolic data. Findings were validated in cohorts of tumors, normal specimens, and cell lines. *In situ* protein expression of SLC6A8 was investigated. Differential gene expression analysis identified a subset of SqCC‐specific genes with metabolic functions through the Reactome database, and/or correlated to specific metabolites through GEMs models. Metabolic profiling identified seven SqCC‐specific metabolites, of which increased creatine levels, in particular, matched to SqCC‐specific expression of *SLC6A8*. Expression of the gene appeared tumor cell‐associated. Elevated creatine levels and overexpression of its transporter SLC6A8 appear a distinct metabolic feature of SqCC. Considering ongoing clinical trials in other malignancies, exploring SLC6A8 inhibition in SqCC appears motivated based on a metabolic addiction hypothesis.

## Graphical abstract

Using multi‐omic characterization, we aimed to identify key regulators specific to squamous cell lung carcinoma (SqCC). SqCC‐specific differentially expressed genes were integrated with metabolics data. High expression of the creatine transporter SLC6A8, along with elevated creatine levels, appeared to be a distinct metabolic feature of SqCC. Further exploration of SLC6A8 inhibition in SqCC is warranted. Created in BioRender. Arbajian, E. (2025) https://BioRender.com/pacsrti.

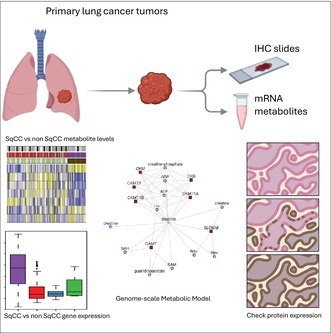

**Abbreviations**
AC: adenocarcinoma
BH: Benjamini–Hochberg
CHKA: choline kinase A
FDR: false discovery rate
FPKM: fragments per kilobase of transcript per million reads
GEM: genome‐scale metabolic model
IHC: immunohistochemistry
LCC: large cell carcinoma
NE: neuroendocrine
NSCLC: non‐small cell lung cancer
NSCLC‐NOS: NSCLC not otherwise specified
OS: overall survival
RNA‐seq: RNA sequencing
SCLC: small cell lung cancer
SLC6A8: solute carrier family 6, member 8
SqCC: squamous cell lung carcinoma
TCGA: The Cancer Genome Atlas
TIC: total ion count
TMA: tissue microarray

## 1 Introduction

Lung cancer is the leading cause of cancer‐related mortality worldwide, with an estimated 1.8 million deaths in 2020 [1]. The disease can broadly be divided into different histological subgroups, including adenocarcinoma (AC), squamous cell carcinoma (SqCC), large cell carcinoma (LCC), large cell neuroendocrine carcinoma, and small cell lung cancer (SCLC). In the current WHO guidelines [2], large cell neuroendocrine carcinoma and SCLC form the neuroendocrine (NE) subgroup, and the remaining histological subgroups form the non‐small cell lung cancer (NSCLC) subgroup.

Extensive genomic profiling has identified a growing number of actionable alterations suitable for inhibition by molecular agents in lung cancer. The most successful applications are found in AC (the most common histological subgroup) and involve the targeting of mutation or fusion gene‐activated protooncogenes like *EGFR*, *ALK*, *ROS1*, and *BRAF* for which molecular diagnostics are now clinical routine [3, 4]. However, these activating mutations are typically not common in other histological subgroups, and targeted agents developed against these alterations are therefore of lesser clinical value outside of AC [5]. Identification of other actionable alterations in, for example, both early and advanced‐stage SqCC (the second most common histological subgroup), is of high clinical value.

In parallel to extensive DNA, RNA, and protein characterization of lung cancer, metabolomic profiling of the disease has also been performed. Importantly, metabolites and their concentrations directly reflect the underlying biochemical activity and state of cells/tissues, and altered tumor cell metabolism has been identified as a hallmark of carcinogenesis [6]. Changes in tumor metabolism involve deregulated metabolism of glucose and amino acids, opportunistic modes of nutrient acquisition, increased nucleotide and polyamine biosynthesis, alterations in metabolite‐driven gene regulation, and metabolic interaction with the tumor microenvironment [6, 7, 8]. Metabolic‐oriented studies in lung cancer have identified biomarkers for early diagnosis, predicted prognosis by comparing changes in metabolites before and after surgery, discovered possible metabolic pathways of the disease, and attempted to determine lung cancer staging [9, 10, 11, 12, 13]. Moreover, recent studies have confirmed that the AC and SqCC histological subgroups appear to be associated with metabolic differences [12, 13, 14, 15, 16].

The aim of the current study was to identify genes and pathways associated with metabolic differences specific to SqCC histology, which might serve as new alternatives for future therapeutic intervention. Based on the integration of matched transcriptional, proteogenomic, and metabolomic data, key genes differentially expressed in SqCC were mapped to metabolite changes through the Human1 genome‐scale metabolic model (GEM). Human1 is a unified GEM–a network‐based tool that collects all known metabolic information of a biological system to computationally describe gene–protein–reaction associations—consisting of 13 417 reactions, 10 138 metabolites (4164 unique), and 3625 genes in the *Homo sapiens* species [17]. We specifically identified creatine metabolism as enriched in SqCC tumors, with associated elevated levels of the creatine transporter *SLC6A8* gene in SqCC tumor cells, indicating specific metabolic fingerprints of lung cancer histologies. Notably, therapeutic inhibition of *SLC6A8* is currently tested in other malignancies, serving as one example of potential new future treatment options in SqCC.

## 2 Materials and methods

### 2.1 Patient cohorts

A multi‐omics tumor discovery cohort consisting of 156 NSCLC patients from our previously reported cohort of 159 (156 NSCLC and 3 SCLC) RNA, DNA, and DNA‐methylation profiled early‐stage lung cancers surgically treated at the Skåne University Hospital in Lund, Sweden between 1989 and 2014 was used to identify genes and metabolites associated with SqCC histology [18]. All patients had early‐stage disease, except for two patients (LU1113 and LU1068) who were treated as operable cases and underwent surgical resection but whose tumors were subsequently diagnosed as stage IV. 73/156 cases had sufficient amounts of fresh‐frozen tumor tissue for metabolic analysis. Histological subgroup classification was performed by a pathologist (HB) according to the WHO 2015 classification [19]. Patient characteristics are summarized in Table 1. In addition to the discovery cohort, we collected a cohort of 27 patients diagnosed with stage IV lung cancer at the Skåne University Hospital in Lund, Sweden, between 2019 and 2020, with tissue material collected through bronchoscopy at the time of diagnosis. Patient characteristics are summarized in Table 2, and this cohort is referred to as the Advanced LUCAS cohort.

**Table 1:** Clinicopathological characteristics of the tumor discovery cohort.

	All cases	AC	SqCC	LCC	LCNEC
Nbr (%)	156 (100%)	102 (65.4%)	30 (19.2%)	10 (6.4%)	14 (9.0%)
Gene expression data	156 (100%)	102 (100%)	30 (100%)	10 (100%)	14 (100%)
Metabolomics data	73 (46.8%)	37 (36.3%)	17 (56.7%)	9 (90%)	10 (71.4%)
Median age (range)	67.9 (33.9–84)	67 (36–83)	72 (59–84)	64.5 (47.6–76.2)	61.1 (33.9–76.9)
Gender (%)					
Female	82 (52.6%)	59 (57.8%)	11 (36.7%)	3 (30%)	9 (64.3%)
Male	73 (46.8%)	43 (42.2%)	18 (60%)	7 (70%)	5 (35.7%)
NA	1 (0.6%)	0 (0%)	1 (3.3%)	0 (0%)	0 (0%)
Smoking status					
Never‐smoker	19 (12.2%)	18 (17.6%)	1 (3.3%)	0 (0%)	0 (0%)
Smoker	111 (71.2%)	71 (69.7%)	25 (83.3%)	5 (50%)	10 (71.4%)
NA	26 (16.6%)	13 (12.7%)	4 (13.4%)	5 (50%)	4 (28.6%)
Stage					
I	119 (76.3%)	82 (80.4%)	26 (86.7%)	3 (30%)	8 (57.2%)
II	26 (16.7%)	13 (12.7%)	3 (10%)	5 (50%)	5 (35.7%)
III	7 (4.5%)	5 (4.9%)	0 (0%)	1 (10%)	1 (7.1%)
IV	2 (1.3%)^a^	0 (0%)	1 (3.3%)^a^	1 (10%)^a^	0 (0%)
NA	2 (1.3%)	2 (2%)	0 (0%)	0 (0%)	0 (0%)
*EGFR* mutation^b^	10 (7.4%)	10 (10%)	0 (0%)	0 (0%)	0 (0%)
*KRAS* mutation^b^	31 (23%)	31 (31%)	0 (0%)	0 (0%)	0 (0%)

^a^ Patients LU1113 and LU1068 were treated as operable cases and underwent surgical resection, but the tumors were subsequently diagnosed as stage IV tumors.

^b^ Percent of patients tested that were positive for the mutation.

**Table 2:** Clinicopathological characteristics of the advanced LUCAS cohort.

	All cases	AC	SqCC	NSCLC‐ NOS	SCLC
Nbr (%)	27 (100%)	15 (55.6%)	6 (22.2%)	3 (11.1%)	3 (11.1%)
Median age (range)	75 (46–95)	77 (55–95)	71 (62–80)	63 (46–72)	78 (77–78)
Gender (%)					
Female	14 (52%)	9 (60%)	3 (50%)	1 (33%)	1 (33%)
Male	13 (48%)	6 (40%)	3 (50%)	2 (67%)	2 (67%)
Smoking status					
Current	7 (26.9%)	5 (35.7%)	1 (16.7%)	0 (0%)	1 (33.3%)
Former	17 (65.4%)	7 (50%)	5 (83.3%)	3 (100%)	2 (66.7%)
Never‐smoker	2 (7.7%)	2 (14.3%)	0 (0%)	0 (0%)	0 (0%)
Stage					
IV A	14 (51.9%)	8 (53.3%)	5 (83.3%)	1 (33.3%)	0 (0%)
IV B	13 (48.1%)	7 (46.7%)	1 (16.7%)	2 (66.7%)	3 (100%)
*EGFR* mutation	3 (11%)	3 (20%)	0	0	0
*ALK* fusion	1 (4%)	1 (7%)	0	0	0

### 2.2 Gene expression data for discovery and advanced LUCAS cohorts

Processed gene expression data for cases in the tumor discovery cohort was obtained from Gene Expression Omnibus (GSE94601) [18].

For the Advanced LUCAS cohort, RNA was extracted from 27 bronchoscopy samples (tissue pieces or fine needle aspirations stored in RNAlater solution (Invitrogen, Carlsbad, CA, USA) directly at the time of clinical investigation), using the Allprep DNA/RNA mini kit (Qiagen, Hilden, Germany). RNA sequencing (RNA‐seq) was performed at the Center for Translational Genomics at Lund University using the Illumina mRNA stranded ligation library preparation protocol and sequenced on a Novaseq instrument (Illumina, San Diego, CA, USA), achieving an average read depth of 22×. Data processing was performed as previously described to generate fragments per kilobase of transcript per million reads (FPKM) expression estimates [20].

### 2.3 Public gene expression and metabolite data

Paired tumor and normal RNA‐seq data from 57 patients with AC and 49 patients with SqCC tumors were obtained from The Cancer Genome Atlas (TCGA). Data was downloaded from the National Cancer Institute's Genomic Data Commons Data Portal (portal.gdc.cancer.gov) in the form of GDS RNAseqv2 FPKM. FPKM data was upper quantile normalized (FPKM‐UQ), followed by log2 transformation using *a* + 1 offset prior to log2 transformation.

Tumor gene expression data for 183 lung tumors was obtained from Djureinovic et al. [21] including 115 AC and 68 SqCC. Gene expression data for 19 144 genes for 185 lung cancer cell lines was obtained from the DepMap portal (www.depmap.org, accessed October 29, 2020). Based on subgroup annotations provided by the repository, the cell lines were divided into 79 AC, 27 SqCC, 10 NSCLC not otherwise specified (NSCLC‐NOS), 17 LCC, and 52 SCLC. From the same repository, we also obtained matched normalized proteomic data for 12 755 proteins for 75 cell lines (37 AC, 12 SqCC, 4 NSCLC‐NOS, 9 LCC, and 13 SCLC). Corresponding metabolite expression for 225 metabolites for 168 cell lines (69 AC, 23 SqCC, 8 NSCLC‐NOS, 17 LCC, and 51 SCLC) was obtained from Li et al. [22].

### 2.4 Genes differentially expressed in SqCC

Genes differentially expressed specifically in SqCC were derived through differential gene expression performed on pre‐processed median‐centered expression data from the discovery cohort [18]. Multigroup comparison was performed by Kruskal–Wallis testing, and genes with Benjamini–Hochberg (BH) adjusted *P*‐value < 0.05 were retained. Paired Wilcoxon tests were then performed for each of these genes and for each subtype combination. Genes that had exactly three tests with BH adjusted *P*‐value < 0.05, all connected to the SqCC histology, were retained as differentially expressed in SqCC only and are from hereon termed SqCC‐specific genes for simplicity. Each of the SqCC‐specific genes was then tested for significance in the Djureinovic cohort [21], using Wilcoxon tests and false discovery rate (FDR) adjusted *P*‐value < 0.05 as a significance cutoff.

### 2.5 Metabolomics

For 73 tumors from the discovery cohort that had sufficient amounts of fresh‐frozen tumor tissue available, 20 to 30 mg of tissue was pulverized via mechanical disruption (TissueLyser II, Qiagen) prior to hydrophilic extraction of intracellular metabolites from tissue using a methanol/acetonitrile/water (50/30/20) with 5 μm d8‐valine extraction solution (250 μL of extraction solution per 10 mg homogenized tissue). Following a 1‐h incubation at −20 °C, the samples were placed in a Thermomixer for 15 min at 4 °C and maximum speed. The samples were then centrifuged for 10 min at maximum speed, and the supernatant was kept at −80 °C in autosampler vials prior to mass spectrometry analysis.

HILIC chromatographic separation of metabolites was achieved using a Millipore (Burlington, MA, USA) Sequant ZIC‐pHILIC analytical column (5 μm, 2.1 × 150 mm) equipped with a 2.1 × 20 mm guard column (both 5 mm particle size) with a binary solvent system. Solvent A was 20 mm ammonium carbonate, 0.05% ammonium hydroxide; Solvent B was acetonitrile. The column oven and autosampler tray were held at 40 °C and 4 °C, respectively. The chromatographic gradient was run at a flow rate of 0.200 mL·min^−1^ as follows: 0 to 22 min: 80% B; 2 to 177 min: linear gradient from 80% B to 20% B; 17 to 17.11 min: linear gradient from 20% B to 80% B; 17.1 to 22.55 min: hold at 80% B. Samples were randomized and analyzed with Liquid chromatography–mass spectrometry in a blinded manner with an injection volume of 5 μL. Pooled samples were generated from an equal mixture of all individual samples and analyzed interspersed at regular intervals within the sample sequence as a quality control.

Metabolites were measured with a Thermo Scientific (Waltham, MA, USA) Q Exactive Hybrid Quadrupole‐Orbitrap Mass spectrometer (HRMS) coupled to a Dionex Ultimate 3000 UHPLC. The mass spectrometer was operated in full‐scan, polarity‐switching mode, with the spray voltage set to +4.5 kV/−3.5 kV, the heated capillary held at 320 °C, and the auxiliary gas heater held at 280 °C. The sheath gas flow was set to 25 units, the auxiliary gas flow was set to 15 units, and the sweep gas flow was set to 0 units. HRMS data acquisition was performed in a range of m/z =70–900, with the resolution set at 70 000, the AGC target at 1 × 106, and the maximum injection time at 120 ms. Metabolite identities were confirmed using two parameters: (a) precursor ion m/z was matched within 5 ppm of theoretical mass predicted by the chemical formula; (b) the retention time of metabolites was within 5% of the retention time of a purified standard run with the same chromatographic method. Chromatogram review and peak area integration were performed using the Thermo Fisher software tracefinder 5.0 and the peak area for each detected metabolite was normalized against the total ion count (TIC) of that sample to correct any variations introduced from sample handling through instrument analysis. The normalized areas were used as variables for further statistical data analysis. TIC‐normalized data for 139 metabolites and 73 cases was used in subsequent analyses. Raw metabolomics data is available at the NIH Common Fund's National Metabolomics Data Repository (NMDR) website, the Metabolomics Workbench, https://www.metabolomicsworkbench.org where it has been assigned Project ID PR002227. The data can be accessed directly via its Project DOI: https://doi.org/10.21228/M8RR8B.

### 2.6 Immunohistochemical evaluation of SLC6A8 expression

Immunohistochemical (IHC) staining was performed on a prospective cohort including 213 patients with primary lung cancer who underwent surgical treatment at the Skåne University Hospital, Lund between 2005 and 2011. The study included 134 AC, 11 NE tumors, and 68 SqCC cases and has been described previously [23, 24, 25]. In this cohort, one large cell neuroendocrine carcinoma case, 18 AC, and 12 SqCC cases overlap with the tumor discovery cohort.

Tissue microarrays (TMA) were used for IHC analysis. The TMA blocks had three cores of 1 mm in diameter per sample (for rare cases, tumor cells were present on only one or two of the cores). For IHC analysis, 4 μm thick sections were stained for SLC6A8 on a Ventana Benchmark automated staining platform using a rabbit polyclonal antibody (Proteintech, Germany) at a 1 : 200 dilution. The slides were scanned and evaluated using the pathXL software (Philips, The Netherlands). Stained slides were evaluated by two independent observers (DE and MJ) who were blinded to clinical data and patient outcomes; for cases with differences in the scoring between evaluators, a third observer (HB) was consulted, and consensus was reached. In all evaluated cases, there were at least 200 tumor cells, and in the clear majority of cases, more than 1000 evaluable cells. Complete membrane IHC staining of tumor cells, or partial membrane staining when discernible to a single cell, was considered positive. Tumor cells with exclusively cytoplasmic staining were disregarded. Special care was taken to only count positive tumor cells and to exclude other protein‐expressing cells, such as macrophages or respiratory epithelial basal cells. Complementary IHC such as CD68 for macrophages as well as hematoxylin–eosin sections of the cores were available for all cases when needed. Protein expression of SLC6A8 in tumor cells was scored using a four‐graded scale: < 1% stained cells, 1% to 10% stained cells, 11% to 50% stained cells, or > 50% stained cells.

### 2.7 Statistics

Two‐group comparisons were performed using two‐sided Wilcoxon tests with subsequent FDR or BH adjustment, and multigroup comparisons were performed using Kruskal–Wallis tests with BH adjustment, except for the comparison of IHC scores across tumor histologies, which was performed using a chi‐squared test. *P*‐value adjustment was performed using the p.adjust function. Whenever a boxplot is used to illustrate our results, the boxplot elements correspond to: (a) center line = median, (b) box limits = upper and lower quartiles, (c) whiskers = 1.5 × interquartile range. Outliers are displayed. All of these analyses were performed in R (www.r‐project.org).

Functional pathway analysis of biological processes was performed using the enrichGO function from the clusterprofiler Bioconductor package version 4.12 [26], also in r.

Survival analysis was performed using the online KMplotter tool [27] with overall survival (OS) as the clinical endpoint. In KMplotter, SqCC tumors were divided into three equally sized groups based on mRNA levels for investigated genes, and univariate Cox regression using the lowest expressing group versus the highest expressing group was performed to assess prognostic association. The online metaboanalyst version 4.0 tool [28] was used for pathway analysis of metabolite networks using default settings and all compounds in the selected pathways as reference sets. The r piano package version 2.14.0 [29] was used to map metabolites to the genes of interest through the Human1 GEM using human‐gem 1.14.0 [17] Information on human metabolic pathway data was also extracted from the Reactome database version 3.7 release 90, using the Reactome term ‘metabolism’ (Reactome ID: R‐HSA‐1430728) which encompasses 15 first‐tier metabolic pathways further subdivided into 86 pathways involving 2202 protein coding genes (https://reactome.org/PathwayBrowser/#/R‐HSA‐1430728). Mapping and overrepresentation analysis of our genes of interest in the full Reactome pathway database was performed using the online analysis tool on the reactome website (https://reactome.org), with filters for *Homo sapiens* and protein coding entities [30].

### 2.8 Experimental design: replication, randomization, and blinding

No replication of RNA‐seq or metabolomics analyses was performed. Results based on computational analyses of previously available data or experimentally derived data in the primary discovery cohort (the LU cohort) were not replicated; instead, they were validated in independent cohorts for which public RNA‐seq, metabolomic, and/or proteomic data existed. The latter included both data based on primary tumor tissue as well as *in vitro* cultured lung cancer cell lines. Patients were not randomized into any groups; group definitions used were based on histopathological subtype or molecular characteristics. Blinding was not applicable to this study. This study is based on defined groups for which statistical comparisons were performed. The sequencing facilities were, however, blinded to the patient and group id, but once data was collected and processed by the investigators, blinding was not applicable.

### 2.9 Ethics statement

The study was approved by the Regional Ethical Review Board in Lund, Sweden (Registration no. 2004/762, 2008/702, and 2014/748) and performed in adherence to the Declaration of Helsinki. For patients included starting in 2004 and onwards, the experiments were undertaken with the understanding and written consent of each subject. For the minority of patients who were included before 2004, specific written informed consent was not required, by decision of the Ethical Review Board and as no sensitive data were used for this study. In accordance with the decision of the Ethical Review Board, information about the study was available for all patients through local advertisements in news media in the region.

## 3 Results

### 3.1 SqCC‐specific gene expression to identify potential metabolic associations

**Fig. 1:**
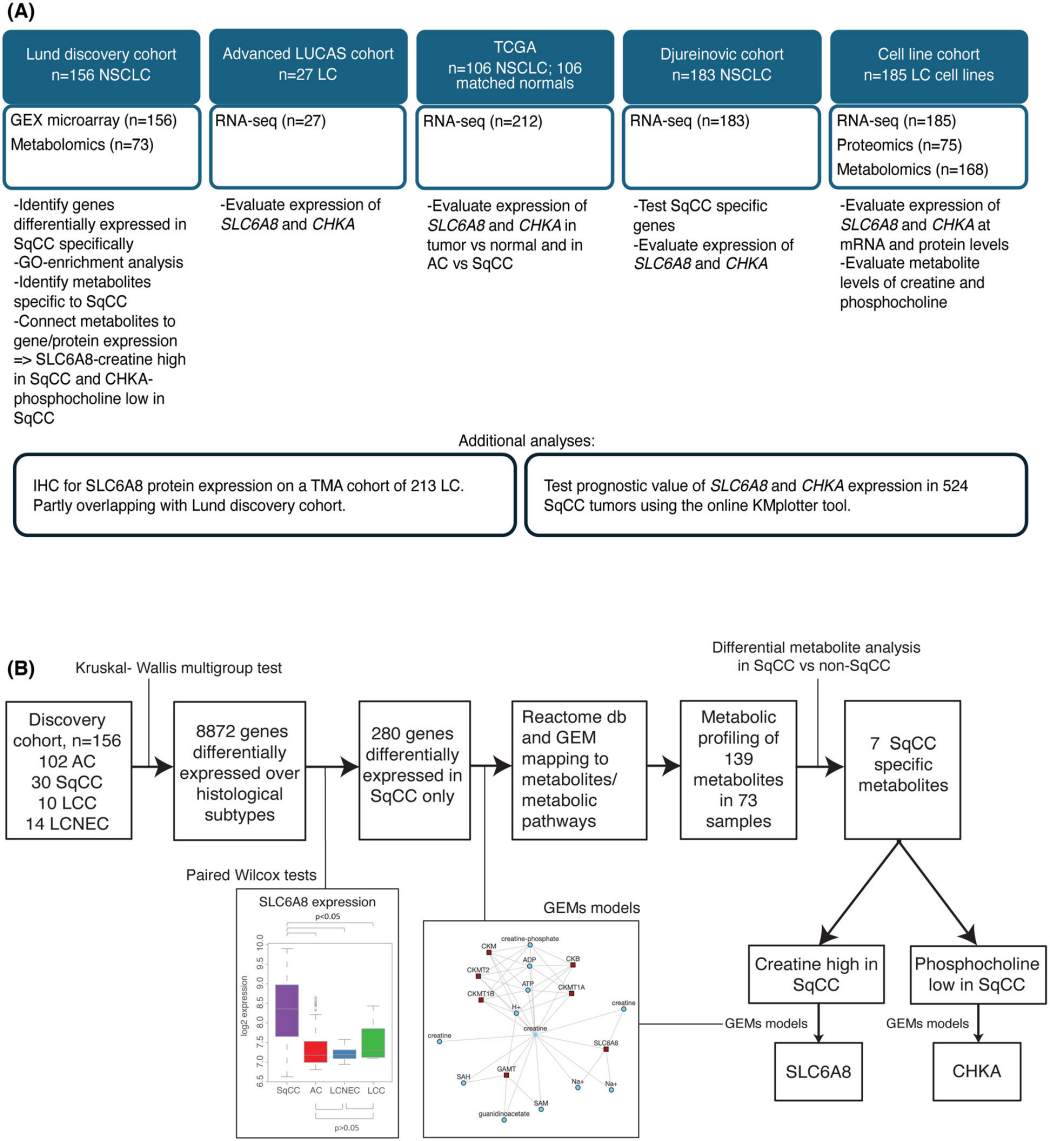
Overview of the study. (A) Overview of the study cohorts with their available data levels and analytic strategies. LC, lung cancer. (B) Study workflow with key findings.

An overview of the study cohorts and of the workflow is provided in Fig. 1. Differential gene expression analysis in the discovery cohort identified 280 genes with mRNA expression specific to SqCC, that is, significantly differentially expressed (BH adjusted *P*‐value < 0.05) between SqCC and each of the other tumor histological subtypes, while not significantly differentially expressed pairwise between the other histological subtypes (Table S1). Of the 280 SqCC‐specific genes, 212 were present in the RNA‐seq data from Djureinovic et al. [21] and of these, 194 genes were also significantly differentially expressed (FDR‐adjusted Wilcoxon *P*‐value < 0.05) in this cohort. GO enrichment analysis of the 280 genes identified 66 enriched biological processes (BH adjusted *P*‐value < 0.05), of which several were expected considering upregulation in SqCC of numerous keratin genes like *KRT6A*, *KRT17*, *KRT16*, and *KRT15*, including tissue development (GO:0009888, adjusted *P* = 0.00003), epidermis development (GO:0008544, adjusted *P* = 0.0002), epithelial cell differentiation (GO:0030855, adjusted *P* = 0.0005), and keratinocyte differentiation (GO:0030216, adjusted *P* = 0.001) (Table S1). No apparent direct associations with metabolic pathways were identified, despite reported metabolic differences between histological lung cancer subtypes [12, 13]. The latter is most likely due to only a single/few key genes in a metabolic pathway, for example, a transporter protein, showing sufficiently large fold‐changes in global differential gene expression analysis. To more comprehensively search for metabolic associations of the 280 SqCC‐specific genes, we accessed information on metabolic pathways in the Reactome database and in the Metabolic Atlas database. First, we extracted information on all human metabolic pathways from the Reactome database, including 15 curated first‐tier metabolic pathways involving 2202 unique protein coding genes. Of the 280 SqCC‐specific genes, 33 were identified as metabolic genes by this approach. The 33 genes mapped to a total of 274 Reactome pathways, but consistent with results from the GO‐enrichment analysis, there was no significant overrepresentation of genes in any pathway (Table S1). We also used human GEMs from the Metabolic Atlas database to map the SqCC‐specific genes to metabolites. Notably, 57 of the 280 SqCC‐specific genes were associated with reactions involving 324 unique metabolites through GEMs (Table S1).

### 3.2 Metabolic profiling of lung cancers identifies two metabolites specific to SqCC

**Fig. 2:**
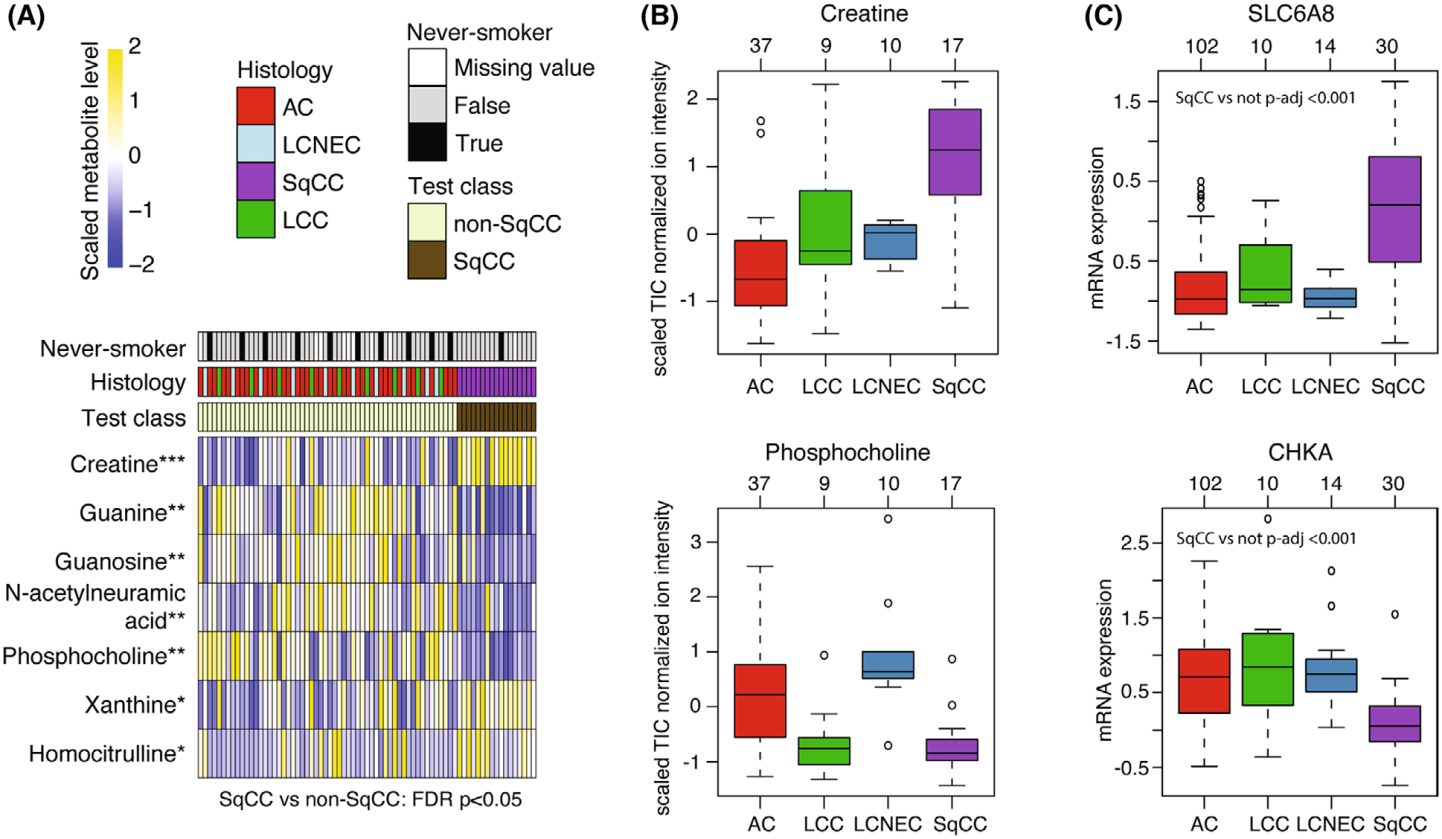
SqCC‐specific metabolites and matching gene expression in lung cancer cases from the discovery cohort. (A) Ordered heatmap of scaled metabolite data for metabolites found to be different in abundance between squamous cell lung carcinoma (SqCC) and non‐SqCC cases in 73 tumors (FDR‐adjusted two‐sided Wilcoxon's *P*‐value < 0.05). ***P ≤ 0.001; **0.001 < *P* ≤ 0.01; *0.01 < *P* ≤ 0.05 (B) Scaled creatine and phosphocholine metabolite levels versus histological subtypes (*n* = 37 adenocarcinoma AC, *n* = 9 large cell carcinoma LCC, *n* = 10 large cell neuroendocrine carcinoma LCNEC, *n* = 17 SqCC).^1^ (C) mRNA expression of *SLC6A8* and *CHKA* associated with creatine and phosphocholine, respectively, across the histological subtypes (*n* = 102 AC, *n* = 10 LCC, *n* = 14 LCNEC and *n* = 30 SqCC).^1^ ^1^The central line in each box indicates the median, the box denotes the interquartile range (IQR) (25th–75th percentiles), and whiskers extend to the data points within 1.5 × IQR from the box limits. Individual points beyond the whiskers represent outliers. Sample sizes for each group are indicated above the boxes.

To investigate the connection between the identified SqCC‐specific genes and relative metabolic patterns in early‐stage lung cancer, we performed mass spectrometry‐based metabolic profiling of 73 tumors from the tumor discovery cohort targeting 139 metabolites (Table S2). Based on a one‐vs.‐rest approach (SqCC vs. non‐SqCC), seven metabolites (creatine, guanine, guanosine, *N*‐acetylneuramic acid, phosphocholine, xanthine, and homocitrulline) were identified as having significantly different levels (FDR‐adjusted Wilcoxon *P*‐value < 0.05) in SqCC vs. non‐SqCC tumors (Fig. 2A,B).

Of the seven significant metabolites, two, creatine (elevated in SqCC) and phosphocholine (decreased in SqCC), involved genes (the creatine transporter gene *SLC6A8* and the choline kinase A gene *CHKA*) also found to have SqCC‐specific mRNA expression (Table S1). Notably, the mRNA expression of both *SLC6A8* and *CHKA* matched the corresponding metabolite level in the tumor tissue (Fig. 2C).

### 3.3 Targeting SLC6A8 and CHKA associated with SqCC metabolite‐specific expression

**Fig. 3:**
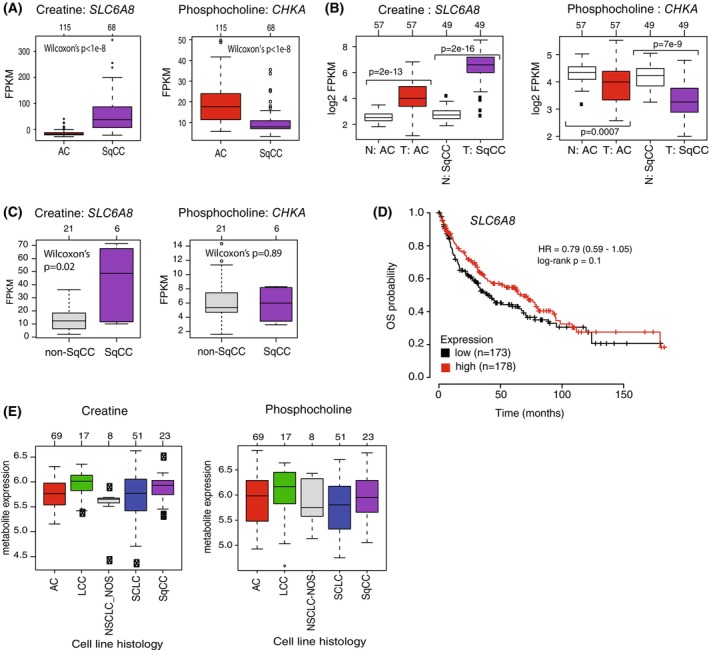
Metabolite abundance of creatine and phosphocholine and associated genes in tumor, matched tumor‐normal tissue, and cancer cell lines. (A) Gene expression of the *SLC6A8* and *CHKA* genes associated with the metabolites creatine and phosphocholine, respectively, in 68 squamous cell lung carcinoma (SqCC) versus 115 adenocarcinoma (AC) tumors from Djureinovic et al. [21] *P*‐values were calculated using a two‐sided Wilcoxon's test and FDR adjusted.^1^ (B) Gene expression of *SLC6A8* and *CHKA* in matched tumor to normal tissue from 57 patients with AC and 49 patients with SqCC from the TCGA consortium. ‘N AC’: matched normal tissue for patients with AC tumors. ‘N SqCC’: matched normal tissue for patients with SqCC tumors. *P*‐values were calculated using a two‐sided Wilcoxon's test and FDR adjusted.^1^ (C) Gene expression of *SLC6A8* and *CHKA* for 6 SqCC versus 21 non‐SqCC stage IV lung tumors.^1^ (D) Kaplan–Meier plot with overall survival (OS) as the clinical endpoint for patients grouped based on *SLC6A8* gene expression divided into two equally sized groups based on mRNA expression. Analysis was performed using the online kmplotter tool. (E) Creatine and phosphocholine metabolite concentrations versus histological subtype in lung cancer cell lines (69 AC, 17 large cell carcinoma LCC, 8 non‐small cell lung cancer not otherwise specified NSCLC‐NOS, 51 small cell lung cancer SCLC, and 23 SqCC).^1^ Metabolite levels were obtained from the study by Li et al. [22]. ^1^The central line in each box indicates the median, the box denotes the interquartile range (IQR) (25th–75th percentiles), and whiskers extend to the data points within 1.5 × IQR from the box limits. Individual points beyond the whiskers represent outliers. Sample sizes for each group are indicated above the boxes.

To substantiate the expression patterns of *SLC6A8* and *CHKA* in lung cancer, we first compared the mRNA expression of the two genes in the Djureinovic et al. RNA‐seq cohort, finding gene expression patterns of both genes closely matching those observed in our discovery cohort (Fig. 3A). Next, we evaluated the gene expression of *SLC6A8* and *CHKA* in matched tumor/normal data for 57 AC and 49 SqCC cases from the TCGA consortium. Again, similar gene expression patterns were observed across the histologies in the TCGA cases compared to our tumor discovery cohort, providing further validation of the latter. Moreover, the mRNA levels of these key genes appeared altered in tumor tissue compared to expression in matched normal tissue, suggesting tumor‐specific expression (FDR‐adjusted Wilcoxon *P*‐values < 0.05 Fig. 3B). To analyze the mRNA expression of the two genes also in advanced lung cancer, we performed RNA‐seq on freshly collected bronchoscopy material (tissue and fine needle aspirations, as outlined [31]) at the time of clinical investigation from 27 stage IV patients (15 AC, 6 SqCC, 3 SCLC, and 3 NSCLC‐NOS) (Table S3). Consistent with the discovery cohort, we observed elevated *SLC6A8* mRNA expression in stage IV SqCC compared to non‐SqCC tumors, while *CHKA* was not significantly expressed (Fig. 3C).

Finally, we tested the prognostic value of *SLC6A8* in 524 primary SqCC tumors using the online kmplotter tool [27], dividing cases into three equally sized groups based on mRNA expression. Based on univariate Cox regression, no significant association with OS was observed for the group with the lowest versus highest mRNA levels (hazard ratio = 0.79, 95% confidence interval = 0.59–1.05; Fig. 3D). Using the same tool, we found similar non‐significant results for *CHKA* (log‐rank *P* > 0.05).

### 3.4 mRNA, protein, and matched metabolite expression of SqCC‐specific SLC6A8 and CHKA in public cancer cell line data

To further investigate SLC6A8 and CHKA in lung cancer, we analyzed mRNA, protein, and metabolite expression of the two genes in lung cancer cell lines. For mRNA and protein patterns, we collected 185 lung cancer cell lines divided into 79 AC, 27 SqCC, 10 NSCLC‐NOS, 17 LCC, and 52 SCLC cell lines from the DepMap consortium. However, gene consistency across histological subgroups of lung cancer cell lines was poor for mRNA and protein expression when compared to the tumor data. *SLC6A8* showed a consistent protein expression pattern, but not mRNA expression pattern, compared to the tumor tissue data, while CHKA did not (Fig. S1).

Li et al. [22] recently reported metabolomic profiling of a large number of cancer cell lines from different organs. In this cohort, we identified 168 lung cancer cell lines divided into 69 AC, 23 SqCC, 8 NSCLC‐NOS, 17 LCC, and 51 SCLC with data for 225 metabolites. However, we did not observe the same patterns of creatine and phosphocholine concentrations as in tumor tissue (Fig. 3E).

### 3.5 SLC6A8 protein expression in early‐stage lung cancer

**Fig. 4:**
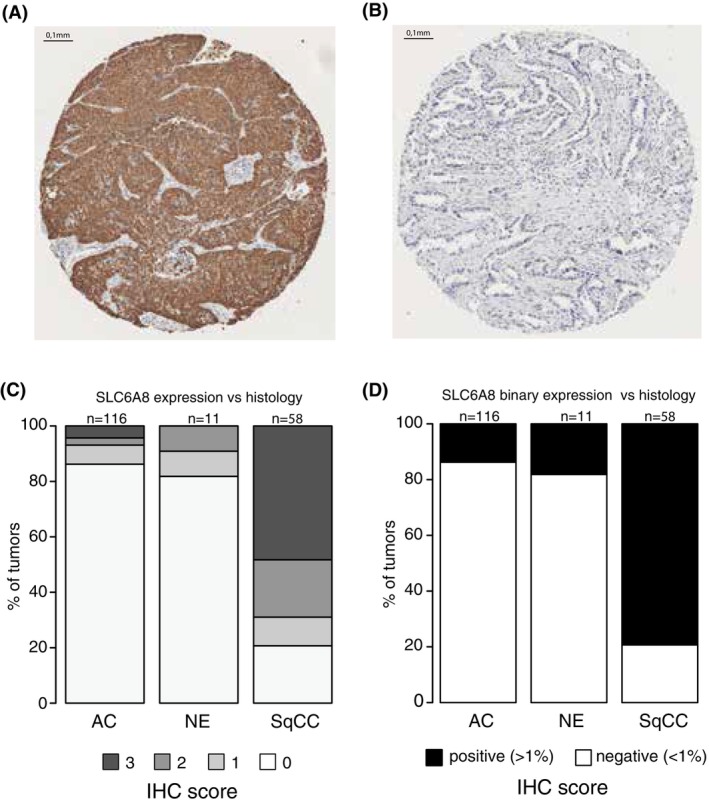
SLC6A8 protein expression versus histological subtypes. (A) SLC6A8 immunohistochemistry (IHC) stain of a squamous cell lung carcinoma (SqCC) tumor with assessed > 50% mean staining. Scale bar: 0.1 mm.^1^ (B) SLC6A8 IHC stain of an adenocarcinoma (AC) tumor with assessed < 1% mean staining. Scale bar: 0.1 mm.^1^ (C) SLC6A8 IHC scores (0 = <1% cells stained, 1 = 1–10% cells stained, 2 = 10–50% cells stained, 3 = > 50% cells stained) versus histological subtype (*n* = 116 AC, *n* = 11 neuroendocrine lung cancer NE and *n* = 58 SqCC).^1,2^ (D) SLC6A8 IHC binary scores (negative < 1% cells stained, positive ≥ 1% cells stained) versus histological subtype (*n* = 116 AC, *n* = 11 NE and *n* = 58 SqCC).^1,2 1^Three cores were stained for each sample and scored by two or three individual pathologists who were blinded to clinical data and patient outcomes. ^2^Chi‐square test *P* < 2e‐16 for all comparisons.

To substantiate protein expression patterns of SLC6A8 in the discovery cohort, we investigated the expression of the gene using IHC in a TMA comprising 213 lung cancers (134 AC, 11 NE, and 68 SqCC tumors) [23, 24, 25]. Protein expression was scored either on a four‐graded scale: < 1%, 1% to 10%, 11% to 50%, or > 50% or as a binary variable (< 1% or ≥ 1%) for the mean IHC score across TMA cores. Consistent with mRNA and metabolite expression patterns in previous cohorts, SqCC tumors showed significantly higher SLC6A8 protein expression than AC and NE tumors (Fig. 4, Chi‐square test *P* < 2e‐16 for all comparisons). Pathologist (DE, HB) evaluation of protein expression established that the protein was primarily expressed in tumor cells. Respiratory basal cells were also expressors, as were some macrophages, although the latter mainly exhibited vague cytoplasmic staining and not the membranous staining that was considered positive. Necrotic debris could manifest with some slight positivity in IHC, but these were mostly easy to disregard.

## 4 Discussion

Metabolic alterations are associated with several pathological conditions, including cancer. Metabolomic profiling of malignancy may thus be a powerful approach to complement findings from other‐omics technologies to improve disease understanding and any molecular phenotypes within. In this study, we searched for metabolite‐connected genes specific to SqCC by integrating transcriptomics, proteomics, and mass spectrometry‐based metabolomic analysis in both tumor tissue and cancer cell lines. In tumor tissue, this approach identified two metabolites, creatine and phosphocholine, specifically associated with SqCC histology, with matched gene and protein expression of key genes included in corresponding metabolic models.

Deregulated creatine (in particular) and phosphocholine metabolite levels between AC and SqCC have been reported previously [12, 13, 32, 33] with similar patterns as observed in this study, but without the link to SLC6A8 or CHKA expression through metabolic models. When comparing bulk tumor tissue to matched normal tissue, the increased mRNA expression of the creatine transporter *SLC6A8* gene and decreased *CHKA* expression appeared tumor‐associated. The latter was further corroborated for SLC6A8 by IHC staining, which showed that *in situ* expression of the SLC6A8 protein was mainly specific to tumor cells (Fig. 4). Moreover, the pattern of significantly higher *SLC6A8* expression specifically in the SqCC histology compared to other lung cancer histological groups was observed also in our cohort of advanced‐stage lung cancers. It is important to acknowledge that our discovery cohort and the advanced lung cancer cohort are not matched for their clinical characteristics. The discovery cohort comprises primary lung cancers from patients who have undergone surgery (stage I to IIIa), whereas the advanced cohort includes only advanced‐stage tumors (stage IIIb to IV). The latter cohort was included in order to validate whether the patterns of SLC6A8 expression hold true also in patients with advanced‐stage disease, which constitute the majority of diagnosed patients. Our findings are in line with previous reports of high SLC6A8 protein expression in NSCLC versus non‐malignant tissue [34, 35]. In the study by Feng et al. [35], based on *in vitro* experiments on NSCLC cell lines representative of both AC and SqCC histologies, it was proposed that SLC6A8 overexpression promotes proliferation, migration, and invasion, accompanied by the activation of the notch signaling pathway, whereas inhibition of SLC6A8 had the opposite effect. Our results indicate that SLC6A8 overexpression is a distinctive feature specifically of SqCC tumor cells in human tumor tissue, that it is highly correlated with increased creatine levels in SqCC tumor tissue compared to other histological subtypes (suggesting a potential difference in tumor metabolism), but, importantly, that these observations replicate poorly in cancer cell line models.

The creatine transporter protein SLC6A8 imports the high‐energy metabolite phosphocreatine into the cell where it can be converted to ATP to fuel the survival of cancer cells as they proliferate and spread. SLC6A8 is also overexpressed in other cancer types, including gastrointestinal cancers where creatine metabolism has been implicated in colon cancer progression and metastatic colonization of the liver [36, 37, 38]. Here, cancer cells may upregulate and release creatine kinase‐B (CKB) into the extracellular space where it generates the high‐energy metabolite phosphocreatine that is transported into the cell by the SLC6A8 transporter protein. Consistent with this finding, genetic depletion of SLC6A8 in colon cancer cell lines significantly reduced cancer growth in animal studies [37]. In ongoing clinical phase 1 and 2 trials, an oral small molecule inhibitor (RGX‐202) of SLC6A8 is tested as a single agent or as combination therapy in patients with advanced gastrointestinal malignancies (ClinicalTrials.gov ID NCT03597581 and NCT05983367, and Kurth et al. [36]). If SLC6A8 inhibition can reduce intracellular levels of phosphocreatine available for ATP synthesis in tumor cells, tumor cell growth and metastasis may potentially be limited. Based on our findings of consistently high creatine levels in SqCC tumor cells compared to other histological subgroups and previous *in vitro* studies suggesting that SLC6A8 overexpression promotes cell invasion in different types of cancers [35, 37, 38], it appears imminent to also investigate the potential of SLC6A8 inhibitors in SqCC treatment. This brings forward an interesting hypothesis of potential metabolite addiction in SqCC, as a parallel to the concept of oncogene addiction in AC based on recurrent oncogenic alterations in different tyrosine kinases.

To ensure reproducibility, we consistently compared our results to different public tumor cohorts and tested our findings also in cancer cell line data. A notable finding was similar patterns of gene expression and/or protein expression in the different tumor cohorts, but not in cancer cell lines. Specifically, congruence between metabolite, mRNA, and protein levels for cell lines as models of SqCC and actual measurements in tumor tissue appeared to be modest, especially for measured metabolite concentrations, an observation reported also by others for 2D cultures of established cell lines [16]. The lack of agreement may be due to different metabolite analysis methods but could also be related to the nature and culture conditions of immortalized human cancer cell lines. Here, the additional validation of key genes in external tumor and normal tissue data, and in data from advanced‐stage lung cancers, provides greater support to the original tumor discovery cohort findings compared to the less supportive cell line data, while also highlighting a potential problem with cell lines as representative *in vitro* models for metabolic changes in tumors (in line with [16]).

Limitations of the current study include the lack of available matched normal tissue in the discovery cohort for matched metabolic profiling. Moreover, the metabolic profiling in the current study is focused on hydrophilic metabolites, thus generating a small set of measured metabolites across all tumors. Moreover, in lung cancer (including SqCC) molecular phenotypes based on gene expression, proteomics, epigenetics, and genetic alterations have been reported [5, 39, 40, 41, 42]. It is plausible, but not yet proven, that such molecular subtypes may also involve changes in cell metabolism within the histological subgroups. Due to the limited size of the current study and the unavailability of matched normal controls, we were not able to account for proposed/potential molecular subtypes in the identification of histology‐associated metabolites. However, for the key finding of the creatine/SLC6A8 connection, the unified high expression in SqCC tumors would argue against large differences within SqCC due to specific molecular phenotypes.

The clinical need for targeted therapy in SqCC is urgent. Current targeted therapies in lung cancer are directed towards genetic alterations in oncogenes rarely found in SqCC tumors [3], leaving advanced‐stage patients the options of immunotherapy and/or chemotherapy. In this study, we demonstrate that the SLC6A8 creatine transporter gene is overexpressed in both early and advanced‐stage SqCC. The generally high levels of creatine in SqCC suggest that it is not a prognostic variable within the subgroup, but rather a subgroup‐specific alteration that may be actionable. This is in line with previous reports of SLC6A8 as a potential prognostic factor in NSCLC in general, but not in SqCC specifically [35]. The most intuitive method to test SLC6A8 inhibition in SqCC would be using cell lines as *in vitro* models. Indeed, Feng et al. did show that overexpression vs silencing of the *SLC6A8* gene in cell lines did increase vs decrease the proliferation and invasion potential of NSCLC cells [35] which corroborates the need for further investigation of the potential of SLC6A8 inhibition in SqCC. However, their study was limited to two cell lines and did not differentiate between the different histological subtypes of NSCLC. Additionally, our analyses of publicly available data from a large number of cell lines covering the different histological subtypes of lung cancer show that there is discordance in metabolite levels and mRNA and protein expression of *SLC6A8* in cell lines as compared to tumor tissue results, thus indicating that cell lines are not the optimal model to fully study the potential of SLC6A8 inhibition as a targeted SqCC treatment and understand the mechanisms related to it. Therefore, alternative *in vitro*, *ex vivo*, or *in vivo* models, such as patient‐derived xenograft or patient‐derived organoid models, which better recapitulate tumor characteristics and heterogeneity would be needed. A main question to answer would be how strong the metabolic dependency is during treatment pressure, that is, whether tumor cells can easily adapt and change their metabolism to overcome inhibition.

In summary, based on integrated analysis of high‐dimensional transcriptomic and proteogenomic data with metabolite profiling, we identified two metabolites in SqCC with associated gene and/or protein expression of key metabolite network genes, linking previous findings from studies in the field into a coherent metabolite‐gene framework and raising a speculation about a potential metabolic addiction in SqCC to be explored *in vivo*. While our multi‐omics study shows elevated creatine levels and overexpression of the associated transporter protein SLC6A8 in primary SqCC tumor tissue, the source behind the actual increased creatine levels in patient tumor tissue remains to be further investigated. Interestingly, the elevated levels of creatine and its transporter protein SLC6A8 may be a potential drug target in SqCC based on oral inhibitors already in clinical phase 1 and 2 use in gastrointestinal malignancies.

## 5 Conclusion

We identified two metabolites in SqCC with associated gene and/or protein expression of key metabolite network genes, linking previous findings from studies in the field into a coherent metabolite‐gene framework and raising speculation about a potential metabolic addiction in SqCC to be explored *in vivo*. Interestingly, the elevated levels of creatine and its transporter protein SLC6A8 may be a potential drug target in SqCC.

## Conflict of interest

The authors declare no conflict of interest.

## Author contributions

EA, JS, MP, and CF conceptualized the study. EA, AK, MJ, and FR carried out experiments. CF, DE, HB, MK, and MP provided clinical expertise and/or resources. MA, JS, CF, and EA carried out data curation. EA and JS analyzed the data. EA, JS, CF, MP, and DE acquired funding. All authors were involved in writing the paper and had final approval of the submitted and published version.

## Ethics statement

The study was approved by the Regional Ethical Review Board in Lund, Sweden (Registration no. 2004/762, 2008/702, and 2014/748). The study was performed in adherence with the Declaration of Helsinki.

## Consent for publication

Not applicable.

## Supporting information


**Fig. S1.** SqCC‐specific metabolites and matching gene and protein expression in lung cancer cell lines.
**Table S1.** SqCC‐specific genes and matched metabolites.
**Table S2.** Metabolomics data for the LU discovery cohort and for 168 lung cancer cell lines from Li et al.
**Table S3.** Gene expression data for the Advanced LUCAS and Djureinovic cohorts.

## Data Availability

Raw and TIC‐normalized metabolomics data can be found in Table S2. Raw mass spectrometry for the metabolomics data is available at the NIH Common Fund's National Metabolomics Data Repository (NMDR) website, the Metabolomics Workbench, https://www.metabolomicsworkbench.org where it has been assigned Project ID PR002227. The data can be accessed directly via its Project DOI: https://doi.org/10.21228/M8RR8B. RNA‐seq FPKM counts are available in Table S3. Data from The Cancer Genome Atlas initiative can be retrieved through the Genomic Data Commons Data Portal at https://portal.gdc.cancer.gov. Gene expression and proteomic data for lung cancer cell lines was obtained from the DepMap portal (www.depmap.org). Other data is available from the corresponding publications as cited in the methods sections.
