# Supplementary material for: Multi‐omic profiling of squamous cell lung cancer identifies metabolites and related genes associated with squamous cell carcinoma
Source: Mol Oncol. 2025 Sep 3;19(12):3806–20. doi: 10.1002/1878-0261.70121 (PMC12688162; doi:10.1002/1878-0261.70121)
Supplement: Supplementary file 1 — Fig. S1. SqCC‐specific metabolites and matching gene and protein expression in lung cancer cell lines. Table S1. SqCC‐specific genes and matched metabolites. Table S2. Metabolomics data for the LU discovery cohort and for 168 lung cancer cell lines from Li et al. Table S3. Gene expression data for the Advanced LUCAS and Djureinovic cohorts. [file MOL2-19-3806-s001.zip › Supplementary files legends.docx]

**Supplementary files legends:**

**Supplementary Figure S1.** **SqCC-specific metabolites and matching gene and protein expression in lung cancer cell lines.** Expression of matched metabolite (n=69 adenocarcinoma (AC), n=17 large cell carcinoma (LCC), n=8 non-small cell lung cancer not otherwise specified (NSCLC-NOS), n=51 small cell lung cancer (SCLC) and n=23 SqCC; from Li et al.), gene (n=79 AC, n=17 LCC, n=10 NSCLC-NOS, n=52 SCLC and n=27 SqCC), and protein (n=31 AC, n=7 LCC, n=1 NSCLC-NOS, n=7 SCLC and n=10 SqCC for SLC6A8 and n=22 AC, n=8 LCC, n=1 NSCLC-NOS, n=11 SCLC and n=6 SqCC for CHKA) expression of (**A**) creatine and (**B**) phosphocholine in lung cancer cell lines (publicly available data from the DepMap portal). The comparisons squamous cell carcinoma (SqCC) vs other subtypes were performed using two-sided Wilcoxon tests and p-values were Benjamini-Hochberg adjusted.

The central line in each box indicates the median, the box denotes the interquartile range (IQR) (25th–75th percentiles), and whiskers extend to the data points within 1.5 × IQR from the box limits. Individual points beyond the whiskers represent outliers. Sample sizes for each group are indicated above the boxes.

**Supplementary Table S1: SqCC specific genes and matched metabolites.** A. 280 squamous cell lung carcinoma (SqCC) specific genes in the LU discovery cohort. B. Pathway enrichment analysis of biological processes for the 280 SqCC specific genes. C. Metabolites (all metabolites from the human genome-scale metabolic model (GEMs) metabolites or 139 measured metabolites) matched to 280 SqCC specific genes through GEMs. D. Mapping and overrepresentation analysis of 33 metabolic genes to all reactome pathways.

**Supplementary Table S2: Metabolomics data for the LU discovery cohort and for 168 lung cancer cell lines from Li et al.** A. Total ion count (TIC) normalized ion intensity of 139 measured metabolites in 73 lung cancer samples B. Raw data ion intensity of 139 measured metabolites in 73 lung cancer samples C. 225 measured metabolites in 168 lung cancer cell lines from Li et al. [23]

**Supplementary Table S3: Gene expression data for the Advanced LUCAS and Djureinovic cohorts.** A. FPKM RNA-seq data for the Advanced LUCAS cohort. B. 194 squamous cell lung carcinoma (SqCC) specific genes also significant in SqCC vs adenocarcinoma (AC)  in the Djureinovic cohort.
