# Supplementary material for: Multi‐omic profiling of squamous cell lung cancer identifies metabolites and related genes associated with squamous cell carcinoma
Source: Mol Oncol. 2025 Sep 3;19(12):3806–20. doi: 10.1002/1878-0261.70121 (PMC12688162; doi:10.1002/1878-0261.70121)
Supplement: Supplementary file 1 — Fig. S1. SqCC‐specific metabolites and matching gene and protein expression in lung cancer cell lines. Table S1. SqCC‐specific genes and matched metabolites. Table S2. Metabolomics data for the LU discovery cohort and for 168 lung cancer cell lines from Li et al. Table S3. Gene expression data for the Advanced LUCAS and Djureinovic cohorts. [file MOL2-19-3806-s001.zip › SupplementaryFigure_1.pdf]

A)

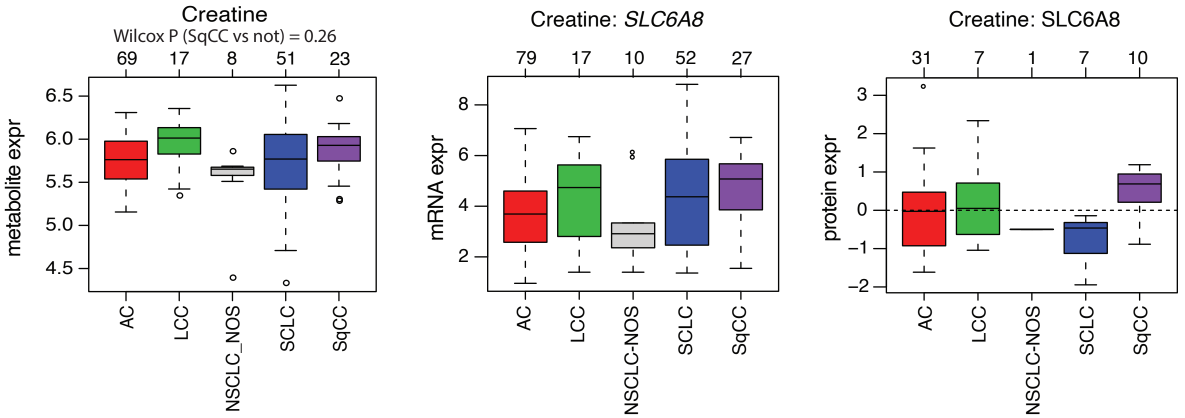

B)

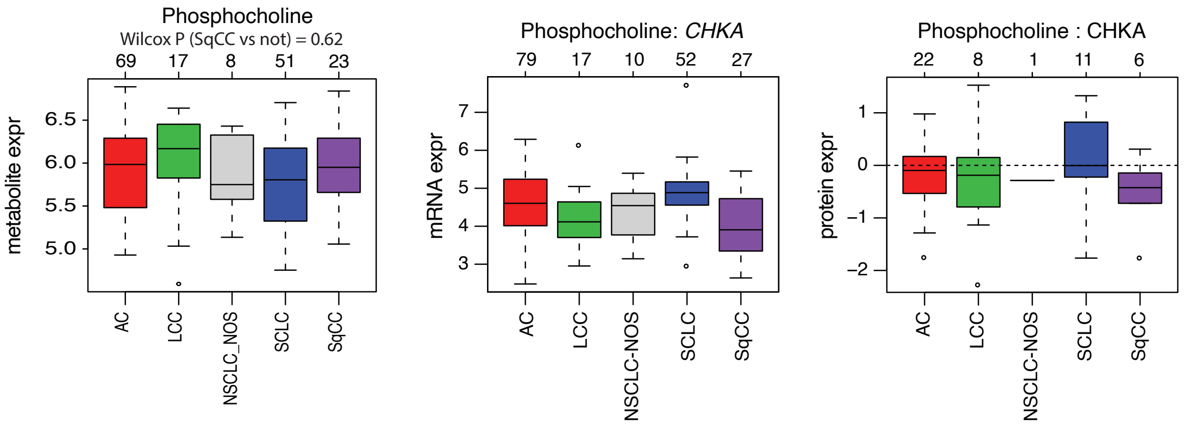

Supplementary Figure S1. SqCC-specific metabolites and matching gene and protein expression in lung cancer cell lines.
